# Polyomavirus JCPyV infrequently detectable in adenoid cystic carcinoma of the oral cavity and the airways

**DOI:** 10.1007/s00428-019-02617-6

**Published:** 2019-07-01

**Authors:** Hanna Hämetoja, Jaana Hagström, Caj Haglund, Leif Bäck, Antti Mäkitie, Stina Syrjänen

**Affiliations:** 1 Department of Oral Pathology, University of Turku and Turku University Hospital, Turku, Finland; 2 Department of Pathology, University of Helsinki and Helsinki University Hospital, Helsinki, Finland; 3 Research Programs Unit, Translational Cancer Biology Program, University of Helsinki, Helsinki, Finland; 4 Department of Surgery, University of Helsinki and Helsinki University Hospital, Helsinki, Finland; 5 Department of Otorhinolaryngology - Head and Neck Surgery, University of Helsinki and Helsinki University Hospital, Helsinki, Finland; 6 Division of Ear, Nose and Throat Diseases, Department of Clinical Sciences, Intervention and Technology, Karolinska Institutet and Karolinska University Hospital, Stockholm, Sweden; 7 Research Programme in Systems Oncology, Faculty of Medicine, University of Helsinki, Helsinki, Finland

**Keywords:** Adenoid cystic carcinoma, Polyomavirus, Oral cancer, Oncogenes, Human papillomavirus

## Abstract

Our objective was to assess the presence of three polyomaviruses, namely SV40, JCPyV, and BKPyV, and human papillomaviruses (HPV) in adenoid cystic carcinomas (ACC) of the minor salivary glands (MiSG) in the head and neck region. The study comprised 68 MiSG ACC patients operated during 1974–2012 at the Helsinki University Hospital (Helsinki, Finland). Medical records and 68 histological samples were reviewed. Polyomaviruses were detected with quantitative PCR and the DNA-positive samples were further analyzed for the presence of viral tumor T antigen (T-ag) with immunohistochemistry. HPV genotyping was performed with a Multiplex HPV Genotyping Kit. Only JCPyV DNA was found in ACC samples, being present in 7 (10.3%) out of the 68 samples. The viral load of JCPyV was low varying between 1 to 226 copies/μg DNA. The JCPyV-positive samples originated from trachea (two samples), paranasal sinuses (one), and oral cavity (two). Additionally, JCPyV positivity was found in one lung metastasis of a tracheal tumor and one local disease failure of an oral cavity tumor. Three JCPyV DNA-positive samples showed weak nuclear staining for large T-ag. In conclusion, only JCPyV but not SV40, BKPyV, or HPV was found in ACC from the upper and lower airways. JCPyV copy numbers were low which might support its role as a “hit and run agent” in ACC carcinogenesis.

## Introduction

Adenoid cystic carcinoma (ACC) is a rare malignancy of glandular structures, which most commonly (70%) appears in salivary glands [1]. Minor glands (MiSGs) are involved more often than major glands (MaSGs) [1, 2]. Etiopathogenesis for ACC as well as for many other salivary gland tumors is still unknown. In ACC, genetic studies have found MYB/NFIB translocations and this fusion oncogene is overexpressed [3]. In addition, studies have provided evidence for Notch-pathway alterations in 11 to 29% of patients [4]. As the pathogenesis of ACC remains unknown, even viral background should be considered. Distinguished viral etiological factors among head and neck cancers are Epstein-Barr virus (EBV) in nasopharyngeal carcinoma and human papilloma virus (HPV) 16 in oropharyngeal squamous cell carcinoma [5, 6].

Furthermore, head and neck cancer studies have gained new knowledge on polyomaviruses (HPyVs) and their coinfection with other viruses [7–11]. A recent study on oncogenic DNA viruses in salivary gland tumors increases the interest on possible role of HPyVs in the pathogenesis of salivary gland tumors [12].

Currently, 13 human HPyVs have been identified [13, 14]. Among them, Merkel cell polyomavirus (MCPyV), BK polyomavirus (BKPyV), and JC polyomavirus (JCPyV) have been associated with human cancers [13]. MCPyV is classified as a grade 2A carcinogen (probable carcinogen) by IARC while BKPyV and JCPyV are grade 2B carcinogens (possibly carcinogenic to humans) [13]. Simian vacuolating virus 40 (SV40) has an ability to cause cancer in animal models but clinical consequences to humans are controversial although SV40 contaminated polio vaccine was used during the years 1955–1963 [13, 15, 16]. HPyVs are small non-enveloped viruses consisting of circular double-stranded DNA genome (5.2 kb) that has two transcriptional units, early and late region [13]. The former encodes large T antigen (T-ag) (90–100 kDA nuclear protein) and the latter, small T-ag (17–22 kDa) viral capsid proteins VP1, VP2, and VP3 [13]. Large T-ag acts similarly as many other known oncoproteins of tumor viruses by interfering with tumor suppressor proteins pRb and p53 [8, 11].

Roughly 80–90% of the population are latent carriers of JCPyV and BKPyV and primary infections are usually manifested subclinically during early childhood [11, 15, 17]. In healthy individuals, secretion of JCPyV and BKPyV might occasionally be detected in urine and saliva [18]. In the case of immunocompromised patients, however, polyomavirus reactivation may cause severe complications. These include polyomavirus-associated nephropathy due to BKPyV reactivation in kidney transplant recipients and JCPyV-related progressive multifocal leukoencephalopathy [15]. HPVs are widely studied and the causality of HPV type 16 and squamous cell carcinoma (SCC) has been classified as grade 1 (IARC vol 100, 2009) [19]. The etiological link between HPV and SCC has specially been shown in cancers of uterine cervix and oropharynx [6, 20]. HPV has also been detected in salivary gland ACC although the connection between HPV and ACC does not seem to be strong [21–23]. According to the “hit and run theory,” different viruses may contribute to tumorigenesis, for instance, polyomaviruses could augment the oncogenic properties of HPV during their co-infections [8].

Altogether, studies on HPyVs, HPVs, and salivary gland tumors are sparse and as far as we know, there are no previous publications on the presence of polyomaviruses in ACC. The aim of this study was to assess the prevalence of the three polyomaviruses, SV40, JCPyV, and BKPyV, and HPV in ACC samples of minor salivary and mucous glands.

## Materials and methods

### Patient and tumor characteristics

Table 1 summarizes the demographic data and tumor characteristics in a retrospective series on 68 patients with MiSG ACC. The patients were diagnosed and treated at the Department of Otorhinolaryngology – Head and Neck Surgery, Helsinki University Hospital (Helsinki, Finland) between 1974 and 2012. The referral area for this tertiary care center currently is 1.6 M. The clinical data of this series have been characterized in our previous study [24] and the Ki67 immunostaining results collected from the same hospital data were now added. The tumor samples were re-evaluated and validated according to the diagnostic criteria by the classification of the World Health Organization classification (both 2005 and 2017) [3, 25]. The present tumor, node, metastasis (TNM) classification does not include tracheal tumors, and thus, the TNM classes on six (8.8%) tracheal ACCs are not given. Additionally, in four cases (5.9%), the TNM class could not be defined.

**Table 1:** Characterization of the 68 patients with an adenoid cystic carcinoma of minor salivary and mucous glands

	*N*	%
Sex		
Male	29	42.6
Female	39	57.4
Female/male ratio	1.35	
Median age, years (range)	58 (24–88)	
Tumor site		
Oral cavity	41	60.3
Paranasal cavities	6	8.8
Trachea	6	8.8
Nasopharynx	5	7.4
Oropharynx	3	4.4
Ear	4	5.9
Larynx	2	2.9
Esophagus	1	1.5
T class		
T1	18	26.5
T2	12	17.6
T3	6	8.8
T4	22	32.4
N/A	4	5.9
N class		
N0	54	79.4
N1	1	1.5
N2	3	4.4
N/A	4	5.9
Stage		
I	16	23.5
II	12	17.6
III	7	10.3
IV	23	33.8
N/A	4	5.9

*N/A*, not available

TNM and stage classification for 62 tumors (trachea excluded)

Altogether, 68 paraffin blocks of tumor samples were available. Samples included 48 samples from primary tumors and 20 disease failures from 15 patients; altogether, we were able to study samples from 53 patients. In addition, ten normal salivary gland tissue samples from the same patients were used as normal controls.

The study comprised MiSG and mucous excreting gland samples from the head and neck area including trachea and esophagus. These gland types in the upper gastrointestinal tract and in the respiratory tract have similar structure and function. They maintain the overall moisturizing of the mucosa. MaSGs on the other hand are activated mainly during eating when they produce serous saliva. Due to the location of MiSGs, just beneath the epithelium, different carcinogenic agents and oncoviruses might have easier access to MiSGs compared with MaSGs.

Institutional Research Ethics Board approved the study concept (Dnro 31/13/03/02/2010, 01 February 2010) and Statistics Finland provided the dates and causes of death.

### DNA extraction

#### Formalin-fixed and paraffin-embedded biopsy samples

DNA was extracted from 5-μm-thick deparaffinized sections (1 cm^2^ in total area) with the high salt method [26]. In brief, after deparaffinization, the sections were lysed in lysis buffer (10 mM Tris-HCl, 400 mM NaCl, and 2 mM EDTA, pH 8.2) with proteinase K (200 μg/mL) overnight at + 37 °C. After digestion, proteins were precipitated with saturated NaCl and the DNA with ethanol.

### HPV detection

DNA was amplified with primer sets 1 and 2 from the Multiplex HPV Genotyping Kit® (DiaMex GmbH, Germany). Primer set 1 contains all HPV primers: nine biotinylated forward and three reverse primers for amplifying the HPV types under investigation. Primer set 2 (DNA quality control primers) contains primers for the amplification of a ß-globin gene fragment to verify the amount and the quality of human genomic sample DNA. A negative control contained no genomic DNA to confirm the absence of a contamination in the amplification reactions. Multiplex HPV Genotyping Kit® detects the following 24 low risk (LR)- and high risk (HR) -HPV genotypes: LR-HPV6, 11, 42, 43, 44, and 70 and HR-HPV16, 18, 26, 31, 33, 35, 39, 45, 51, 52, 53, 56, 58, 59, 66, 68, 73, and 82. The labeled hybrids were analyzed with a Luminex LX-100 analyzer (Bio-Plex 200 System, Bio-Rad Laboratories, Hercules, USA).

### Quantitative detection of SV40, JCPyV, and BKPyV

Presence of SV40, JCPyV and BKPyV DNA in the samples was detected by quantitative polymerase chain reaction (qPCR) (Roche, Light Cycler 96) targeting their oncogenic large T-ag as described by McNees and coworkers, with slight modification [15]. RNase P was used as a reference gene (TaqMan® Copy Number Reference Assay RNase, Applied Biosystems, Foster City, CA, USA) to a relative expression of the target genes.

The primers and probes were designed as described earlier [15] and produced by Life Technologies as given in Table 2. The probes for the target genes SV40, JCPyV, and BKPyV were labeled with 6-carboxyfluorescein (FAM) and (VIC) was used for labeling the probe for the reference gene RNase P. The qPCR reactions were performed in 20 μl volume in micro titer plate. The following reaction conditions were used: 900 nM of each primer and 100 nM of their analogous probe, 10 μl of TaqMan® Universal Mix II and 300 ng template DNA. The detection of the reference gene TaqMan® RNase P (Applied Biosystems) was performed according to the manufacturer’s recommendations. The conditions for all qPCR reactions were as follows: 2 min at 50 °C, 10 min denaturation at 95 °C followed by 45 cycles of amplification with 95 °C denaturation for 15 s, and annealing/extension at 60 °C for 60 s. Amplification data measured as an increase in reporter fluorescence were collected in real time and analyzed by the Roche, Light Cycler 96 software.

**Table 2:** Primers and probes for SV40, JCPyV, and BKPyV

Name	Sequence detection
SV40 primer forward	GAT GGC ATT TCT TCT GAG CAA A
SV40 primer reverse	GAA TGG GAG CAG TGG TGG AA
JCPyV primer forward	TTC TTC ATG GCA AAA CAG GTC TT
JCPyV primer reverse	GAA TGG GAA TCC TGG TGG AA
BKPyV primer forward	CTT TCT TTT TTT TTT GGG TGG TGT T
BKPyV primer reverse	TTG CCA GTG ATG AAG AAG CAA
SV40 T-ag probe 5′-FAM	CAG GTT TTC CTC ATTAAA
JCPyV T-ag probe 6 FAM	CCA CTT CTC ATT AAA TG
BKPyV T-ag probe 6 FAM	AGT GTT GAG AAT CTG C

Adapted from McNees et al. 2005

The linear standard curves for JCPyV and BKPyV were obtained with a serial dilution of plasmids with amounts ranging from 1.2*10^2ng/μl to 1.2*10^-2 ng/μl for JCPyV and 9.5*10^0 to 9.5*10^-3 ng/μl for BKPyV. The standards for SV40 detection were constructed with a serial dilution of COS1 cell line DNA (which contains one copy of SV40/cell) with an amount range from 5.0*10^4 to 5.0*10^0cells/μl, while the standards for the reference gene RNase P were acquired with a serial dilution of human placenta DNA extractions (Sigma-Aldrich, Darmstadt, Germany) ranging from 5.09*10^2 to 5.09*10^-2. Cq values of less than 37 were considered positive. The copy numbers were calculated as copies in 1 μg human DNA.

### Immunohistochemistry

Immunohistochemistry was performed for JCPyV-positive samples only as BKPyV and SV40 remained qPCR negative. For immunohistochemistry, there are 4-μm-thick sections of formalin-fixed and paraffin-embedded blocks. The slides were deparaffinized in xylene and rehydrated in a series of ethanol solutions. Endogenous peroxidase activity was blocked by incubation of the slides with 3% hydrogen peroxide for 15 min. Epitope retrieval was performed using boiling in 1 mM citrate buffer, pH 6.0 in the microwave for 5 min, twice. The primary antibody used was a mouse monoclonal anti-simian virus T-ag that cross-reacts with JCPyV T-ag (Anti-SV40 Antibody, clone Pab101, LifeSpan BioSciences Inc., Seattle WA, USA) and the dilution of 1:75 was used. The tissue was incubated with the primary antibody overnight, followed by detection with Dako REAL Detection System, Peroxidase/DAB+, Rabbit/Mouse, Dako, Glostrup, Denmark) and counterstained with hematoxylin.

## Results

### The presence of JCPyV, BKPyV, SV40, and HPV in ACC and in normal salivary gland tissue

All 68 samples remained negative for BKPyV, SV40, and HPV but seven of 68 (10.3%) samples showed JCPyV DNA positivity. The characterization of the seven patients with JCPyV-positive ACC is given in Table 3. The viral load of JCPyV was low in all samples varying from 1 to 226 copies/μg DNA. The locations of JCPyV DNA-positive ACCs were as follows: two in the trachea, one in the lung metastasis of a tracheal tumor, one in the paranasal sinuses (stage IV tumor), two in the oral cavity (stage I and IV tumors), and one in local disease failure of an oral cavity tumor. All the normal salivary gland control tissues showed negativity to JCPyV, BKPyV, and SV40.

**Table 3:** Characterization of the patients with a JCPyV-positive adenoid cystic carcinoma of the minor salivary and mucous glands

Patient	Anatomic subsite	Age	Sex	Smoking	TNM classification	Stage	Treatment	Surgical margins	Growth pattern	Proliferation index Ki67 (%)	Neural invasion	Disease failure	Status	Copy number/1 μg DNA
1	Trachea	43	F	No			Surgery	Positive	Tubular	N/A	No	Distant metastasis in lungs	DOC	1.11
2	Trachea	54	F	No			Surgery	Positive	Cribriform and tubular	35	No		NED	33.95
3	Trachea (lung metastasis)	47	F	No			Surgery	N/A	Cribriform	N/A	N/A	Distant, local, locoregional	DOD	7.18
4	Paranasal sinuses	60	M	Yes	T_4B_N_0_M_0_	IVB	Oncological		Cribriform and tubular	N/A	Yes	Local	DOC	16.51
5	Oral cavity, gum	80	F	N/A	T_1_N_0_M_0_	I	Surgery	Negative	Cribriform	4	No		DOC	225.60
6	Oral cavity, hard palate	59	M	N/A	T_4B_N_0_M_0_	IVB	Surgery and oncological	Positive	Cribriform and solid	30	Yes		DOC	3.25
7	Oral cavity, floor of the mouth (local disease failure)	72	F	N/A	T_4A_N_0_M_1_	IVA	Surgery	Positive	Cribriform	N/A	Yes	Local	DOD	15.47

*N/A*, not available; *TNM*, tumor, node, metastasis; *DOC*, dead of other cause; *NED*, no evidence of disease; *DOD*, dead of disease

### Patients with JCPyV-positive ACC

Table 3 shows the details of the seven patients with JCPyV DNA-positive ACC. The mean age of the patients with JCPyV DNA-positive tumor was 59 years (range, 43–80) and female to male ratio was 2.5. In the whole cohort, the mean age was similar, 58 years (range, 24–88), but the female and male ratio was smaller, being 1.35. Among the JCPyV-positive ACCs, the most common growth pattern was cribriform (42.8%), and the second was a combination of cribriform and tubular (28.6%), followed by tubular (14.3%), and combination of cribriform and solid (14.3%), which simulates the pattern of the whole cohort [24]. Neural invasion (perineural, intraneural, or both) was present in three (42.9%) out of the JCPyV-positive tumors and in 57.4% in the whole cohort. Proliferation index of Ki67 immunostaining varied from 4 to 35% (median 30%) among the JCPyV-positive tumors and from 4 to 80% (median 30%) in the whole cohort.

### Immunohistochemistry versus JCPyV DNA positivity

**Fig. 1:**
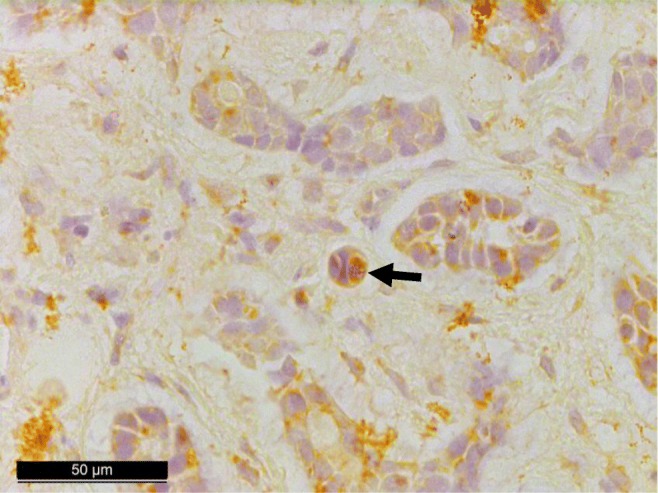
Immunohistochemical staining for large T antigen showing JCPyV positivity in adenoid cystic carcinoma of the trachea (patient #1 in Table 3)

**Fig. 2:**
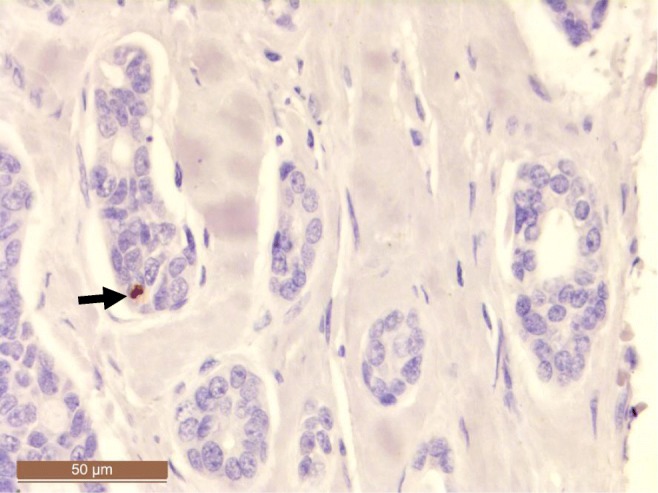
Immunohistochemical staining positivity for large T antigen in adenoid cystic carcinoma of the trachea (patient #2 in Table 3)

In total, three out of the seven JCPyV DNA-positive tumor sections showed weak nuclear immunohistochemistry positivity for large T-ag as presented in Figs. 1 and 2. Copy numbers for two tracheal tumors and one oral cavity tumor were 1.11, 33.95, and 3.25/1 μg DNA, respectively. Immunopositivity had no association with the clinical outcome of the disease.

### Second primary tumors

Of note is that some of the patients with JCPyV DNA-positive ACC suffered from other malignancies as well. One patient with a tracheal ACC with thyroid gland invasion was in addition diagnosed with a papillary microcarcinoma in the thyroid gland. The patient with an early stage I ACC in oral cavity died of SCC of the lungs soon after ACC treatment. Furthermore, the patient with ACC in paranasal sinuses had recovered from lymphoma 6 years earlier.

## Discussion

Our present results show that JCPyV may be found in MiSG ACC samples by qPCR. However, the prevalence of JCPyV in ACC was low, i.e., only 10.3%, and JCPyV presented with low copy numbers. Three out of the seven JCPyV-positive tumors were from the oral cavity while the rest were located in the upper and lower airways. As the number of JCPyV-positive cases was sparse and the viral loads were low, we can only speculate the possible viral role as an etiological factor for ACC. A “hit and run hypothesis” has been proposed for certain oncoviruses. Accordingly, this hypothesis claims that viruses can mediate cellular transformation through an initial “hit,” while maintenance of the transformed state is compatible with the loss (“run”) of viral molecules [27]. The low copy numbers in our study suggest the role of JCPyV in hit and run carcinogenesis model rather than as a continuous driver of the tumor formation. None of the HPyVs were detected in the normal salivary gland control tissue.

We were able to locate large T-ag on nuclei of the tumor cells by using SV40 antibody, which is cross-reacting with both JCPyV and BKPyV. qPCR technique is an accurate method for detecting HPyV DNA, but in the current study, immunohistochemical expression did not correlate well with qPCR findings, while only three out of the seven JCPyV-positive samples stained positively. Possible explanation for the negative staining might be that only few cells in the samples infected with JCPyV are transcriptionally and translationally active. According to the “hit and run theory,” positive signals are detected only from sporadic cells (Figs. 1 and 2). Another possibility is that these approximately 7 μm wide cells might have been cut out from the deeper sections prepared for immunohistochemistry.

The etiological risk factors for all salivary gland malignancies include exposure to nickel compounds and silica dust, employment at rubber manufacturing, hairdressers´, and beauty shops, as well as irradiation, EBV, and HIV infection [10, 28, 29]. At molecular level, germline BRCA mutations and genetic variants in DNA double-strand brake repair genes have been related to the risk for salivary gland cancers including ACC [3].

As far as we know, there are no previous studies on the prevalence of JCPyV or other HPyVs in ACC. As discussed earlier, 80–90% of population are latent carriers of JCPyV and BKPyV and HPyVs have been detected in saliva of healthy individuals, which strengthens the hypothesis of saliva being the transmission route for HPyVs [11, 15, 17, 18]. Thus, the reservoir of HPyVs might be in salivary glands or in oral or oropharyngeal mucosa. An earlier experimental study showed that the injection of polyomavirus into salivary gland tissue of mice resulted in tumor formation resembling that of human pleomorphic adenoma [30]. Further, SV40 DNA has been identified in pleomorphic adenoma of parotid gland [31].

Overall, studies concerning HPyVs in head and neck cancers are rare [7, 9, 11, 32, 33]. Kutsuna et al. have linked high viral JCPyV load to oral SCC of the tongue, but they did not find any prognostic value for JCPyV [11]. Zheng et al. suggested that JCPyV could have an oncogenic role in the squamous cell carcinogenesis of tongue, pharynx, and larynx [32]. Contradictory to their findings, Polz-Gruszka et al. did not find JCPyV in any of the 62 oropharyngeal SCC in their series but found BKPyV in 17.7% of the tumor samples [7]. In these studies, viral DNA was detected by qPCR [7, 11, 32]. Some studies have reported negative HPyV results in head and neck cancer. Palmieri et al. did not detect SV40, BKPyV, or JCPyV DNA by qPCR evaluation in oral cavity SCC [9].

HPVs have been studied in ACC but the association does not seem to be strong [21–23]. However, recently, a new subtype of non-keratinizing SCC has been found in the sinonasal area having features of both ACC and SCC and being usually HPV type 33 positive [3, 34]. This entity is named as HPV-related multiphenotypic sinonasal carcinoma [3, 34]. In our study, HPV-positive ACCs were not detected.

Further studies are thus needed to understand the possible role of HPyV in the carcinogenesis of ACC.

## Conclusions

Only JCPyV DNA but not SV40, BKPyV, or HPV was found in ACC from the upper and lower airways. JCPyV copy numbers were low which might support its role as a “hit and run agent” in ACC carcinogenesis.
